# Aurachins, Bacterial Antibiotics Interfering with Electron Transport Processes

**DOI:** 10.3390/antibiotics12061067

**Published:** 2023-06-17

**Authors:** Sebastian Kruth, Markus Nett

**Affiliations:** Laboratory of Technical Biology, Department of Biochemical and Chemical Engineering, TU Dortmund University, 44227 Dortmund, Germany; sebastian.kruth@tu-dortmund.de

**Keywords:** aurachin, quinolone antibiotics, antibacterial, antiprotozoal, respiratory chain, cytochrome *bd*, biotechnological production

## Abstract

Aurachins are farnesylated quinolone alkaloids of bacterial origin and excellent inhibitors of the respiratory chain in pro- and eukaryotes. Therefore, they have become important tool compounds for the investigation of electron transport processes and they also serve as lead structures for the development of antibacterial and antiprotozoal drugs. Especially aurachin D proved to be a valuable starting point for structure-activity relationship studies. Aurachin D is a selective inhibitor of the cytochrome *bd* oxidase, which has received increasing attention as a target for the treatment of infectious diseases caused by mycobacteria. Moreover, aurachin D possesses remarkable activities against *Leishmania donovani*, the causative agent of leishmaniasis. Aurachins are naturally produced by myxobacteria of the genus *Stigmatella* as well as by some *Streptomyces* and *Rhodococcus* strains. The recombinant production of these antibiotics turned out to be challenging due to their complex biosynthesis and their inherent toxicity. Recently, the biotechnological production of aurachin D was established in *E. coli* with a titer which is higher than previously reported from natural producer organisms.

## 1. Introduction

Natural products are an essential resource for drug development. From the past until today, many bioactive natural products with anticancer, antifungal, antibacterial, and antiprotozoal effects have been used directly as medicine or have been the inspiration for lead structures in drug development [1,2,3]. Among these privileged structures are alkaloids featuring a quinoline or quinolone scaffold. Examples include the antiplasmodial quinine, the antitumor agent camptothecin, or the Pseudomonas Quinolone Signal (PQS) [4,5,6]. In particular, quinine is an outstanding example since it can be viewed as the starting point of quinoline-based drug discovery and development [7]. 

Several natural products featuring a quinoline or quinolone motif were extracted from plants, especially rutaceous plants [4,5]. Nevertheless, microorganisms also represent excellent resources for the discovery of such molecules, as exemplified by the aurachins [6]. These natural products constitute a large family of prenylated quinoline alkaloids, which were first described from the myxobacterium *Stigmatella aurantiaca* [8]. Later, the aurachins were also isolated from a related species, *Stigmatella erecta*, as well as from selected strains of the actinomycete genera *Rhodococcus* and *Streptomyces* [9,10,11,12]. Very soon it was realized that the aurachins possess potent antibiotic, as well as other pharmaceutically relevant properties [8,13,14,15]. 

This review provides, for the first time, a comprehensive overview of the diverse biological activities of these quinoline antibiotics, which have become important lead structures for the development of drugs against infectious diseases. The different biosynthesis routes to aurachins in myxobacteria and actinomycetes are presented. Furthermore, we describe and discuss the biotechnological production and derivatization of aurachins.

## 2. Biological Activities

Aurachins were originally discovered due to their antimicrobial properties. In a paper disc assay, they showed activities against Gram-positive bacteria and, in higher concentrations, also against some fungi. Their structural relatedness to 2-heptyl-4-hydroxyquinoline-*N*-oxide (HQNO), a known electron transport inhibitor, suggested early on that the aurachins might have the same cellular target [8]. In several subsequent studies, this assumption was confirmed. Not only were the aurachins found to affect the thylakoid membrane’s photosystem II and cytochrome *bf* complex, but they were also shown to block the mitochondrial and bacterial NADH:ubiquinone oxidoreductase (respiratory complex I) and the cytochrome *bc*_1_ complex (respiratory complex III) [8,16,17]. Furthermore, it was demonstrated that the mitochondrial membrane potential in human U-2 OS osteosarcoma cells is lowered by aurachin D (**1**) [14]. Bioactivity testing revealed cytotoxic effects of **1** on various mammalian cell lines, including human cancer cell lines [14,18]. 

**Figure 1:**
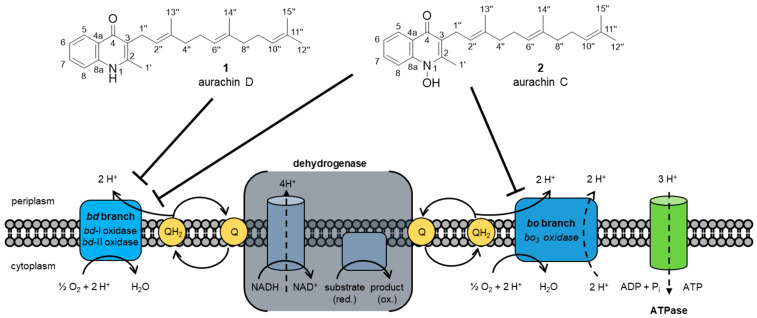
Schematic representation of the inhibition of the different terminal oxidases by aurachin D (**1**) and aurachin C (**2**) in *E. coli*. The *bd* branch contains two terminal oxidases (*bd*-I, *bd*-II), while the *bo* branch contains one terminal oxidase (*bo*_3_). Quinones (Q) are reduced by the enzymatic reaction of the dehydrogenases with two electrons to quinols (QH_2_). The three terminal oxidases oxidize Q to QH_2_ at their oxidation site. The released electrons are transferred to the respective active site of the enzymes. In the active site, molecular oxygen is then reduced to water. Compound **2** inhibits the oxidation site of all terminal oxidases, whereas **1** only inhibits the oxidation site of the *bd*-type oxidases [19,21,22,24].

Since the 1990s, the effects of the aurachins on the respiratory chain of *Escherichia coli* were intensively researched [19,20]. While **1** is a highly selective inhibitor of the terminal oxidase cytochrome *bd* of *E. coli*, aurachin C (**2**) also affects the terminal oxidase cytochrome *bo*_3_ (Figure 1). Both aurachins inhibit the reduction of quinol to the corresponding quinone species at the quinol oxidation site of *bd* [19,21]. The replacement of the prenyl moiety in **1** and **2** with aliphatic side chains of different lengths revealed that only their quinolone ring structure is essential for this inhibition [19,22]. *E. coli* contains two different cytochrome *bd* oxidases. In contrast to *bd*-II, *bd*-I is well characterized. Recently, the 3.0 Å resolution structure of *E. coli bd*-II with bound **1** was reported [21]. The natural product was found to bind in a hydrophobic pocket formed by the Q-loop and transmembrane helices 6 and 7 of AppC (subunit of *bd*-II). The Q-loop is a periplasmic domain responsible for binding and oxidizing quinol [23]. The binding of **1** stabilized the Q-loop and allowed the full characterization of the complete Q-loop [21]. 

Noteworthy, cytochrome *bd* is exclusive to the respiratory chain of prokaryotes, and its upregulation is linked to the virulence and drug resistance of pathogenic bacteria [25]. Therefore, **1** represents an interesting antibacterial lead structure. A structural derivative of **1** named AD7-1, which features a heptyl side chain instead of a prenyl side chain, combines high selectivity and potent inhibition of cytochrome *bd*-I. It was, therefore, promoted for further studies on a physiological level [22]. 

Unlike *E. coli*, mycobacteria possess a respiratory chain similar to the mitochondrial chain containing cytochrome *c*. However, their respiratory chain is branched, including a second chain with a cytochrome *bd* terminal oxidase [26]. Bioactive compounds that inhibit both branches are urgently sought. Especially for treating *Mycobacterium tuberculosis* infections, **1** was postulated as a starting point for developing new combination therapies [26,27]. Structure-activity relationship (SAR) studies indicated that both the farnesyl side chain of **1** and a citronellyl side chain exhibit the optimal length for inhibiting *M. tuberculosis* cytochrome *bd* oxidase. Additionally, the substitution of the aromatic ring of **1** at position 6 with fluorine was tolerated. All three compounds were marked as promising drug candidates for further inhibition studies [27]. 

The sodium-translocating NADH:ubiquinone oxidoreductase (Na^+^-NQR) represents another novel target that is strongly inhibited by **1**. Not only is Na^+^-NQR the major Na^+^ pump in pathogens such as *Vibrio cholera*, but it is also absent in eukaryotic cells [28]. Moreover, Na^+^-NQR is the first enzyme in the respiratory chain of *V. cholerae*. The quinolone **1** and two structural analogs featuring a photoreactive azido group, PAD-1 and PAD-2, were used to identify the ubiquinone binding site [29]. It was demonstrated that these compounds bind to the cytoplasmic surface of the NqrB subunit of Na^+^-NQR. Furthermore, there is a partial overlap between the binding site of ubiquinol and the inhibitors within the interfacial region of NqrA and NqrB, located near the cytoplasmic surface [29,30]. 

Aurachins, especially **1** and **2**, have proven to be valuable test compounds to study the respiratory chain of different bacteria. Although these antibiotics were shown to inhibit vital pathways in pathogenic bacteria, no or only weak antibacterial activities were reported when the aurachins were tested in in vivo assays [8,14,27]. Therefore, the delivery of the aurachins to the cytoplasmic-membrane-bound targets represents a challenge that must be still overcome [27]. 

Besides their antibacterial activities, the aurachins also exhibit antiprotozoal properties [13,14,15]. This discovery was not unexpected, since many antiprotozoal compounds are quinoline derivatives [31]. The activity for **1** was demonstrated in vitro against multiple *Plasmodium falciparum* strains [13,14,15]. *P. falciparum* is the most dangerous *Plasmodium* species and can cause cerebral malaria [31]. Although **1** did not outperform the antiplasmodial drug chloroquine [13,15], it was efficient against a chloroquine-resistant strain [14]. The lack of cross-resistance suggests a different mode of action for **1** and chloroquine, but it is also possible that **1** is no substrate of the mutated *P. falciparum* Chloroquine Resistance Transporter (PfCRT) [32]. In in vitro assays, **1** showed only moderate activities against different subspecies of *Trypanosoma brucei,* the causative agent of African sleeping sickness [14,15]. In stark contrast, **1** exhibited superior activities and a broad therapeutic window against *Trypanosoma cruzi* and *Leishmania donovani*, which cause Chagas disease and visceral leishmaniasis, respectively [15]. The same study also revealed that minor variations of the aromatic ring of **1** can alter its antiprotozoal effects. Up to now, the respective biological target of the naturally derived aurachins in *Plasmodium*, *Leishmania* and *Trypanosoma* spp. has not been identified. It is noteworthy, however, that a structural derivative of **2** was found to target the type II NADH dehydrogenase (NDH2) of malaria parasites, which is absent in mammalian mitochondria [33].

The biological function of aurachins for the respective producers remains unclear. For *Rhodococcus* or *Streptomyces* strains, aurachins may act against other competing microorganisms. Because the myxobacterium *S. aurantiaca* Sg a15 also produces two other classes of respiratory chain inhibitors in addition to the aurachins, it was assumed that this bacterium has evolved a strategy to prevent resistance development in the target organisms [34]. Similar to other myxobacteria, *S. aurantiaca* is a predatory myxobacterium and it is, therefore, possible that the aurachins support the killing of other microorganisms for their consumption [35,36]. 

## 3. Aurachin Biosynthesis

**Figure 2:**
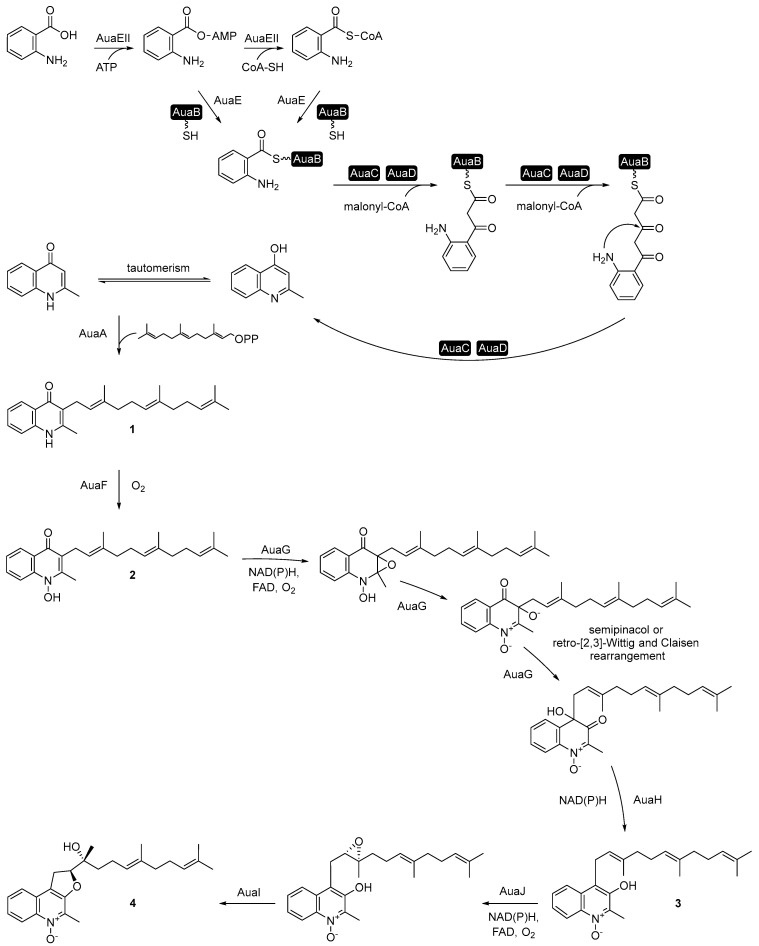
Biosynthetic pathway from anthranilic acid to aurachin A (**4**) in *S. aurantiaca* Sg a15. Black-boxed enzymes are constituents of a type II PKS.

Feeding studies with isotopically labeled precursors in the *S. aurantiaca* strain Sg a15 revealed that the quinolone scaffold in aurachins originates from anthranilic acid [34]. Its biosynthesis involves a type II polyketide synthase (PKS) [37,38]. Compared to the type II fatty acid synthase of bacteria and plants, type II PKSs comprise several individual enzymes. At least two keto synthase units (KSα and KSβ) and an acyl carrier protein (ACP) are part of the so-called minimal PKS [39]. In *S. aurantiaca* Sg a15, aurachin biosynthesis starts with the loading of anthranilic acid onto the ACP AuaB. This reaction is catalyzed by the anthranilate-CoA ligase AuaEII and the anthranilate-CoA:ACP acyltransferase AuaE (Figure 2). AuaEII is responsible for the formation of the anthranilate-CoA thioester and AuaE for the substrate transfer to AuaB. AuaE also accepts the anthranilate-AMP intermediate of AuaEII as a substrate, though the preferred substrate is anthranilate-CoA [40]. Iterative decarboxylative condensation steps of malonyl-CoA extender units with the acyl starter usually follow the loading of the starter unit of a type II PKS. While the keto synthase units of a minimal PKS catalyze the condensation reaction, the ACP serves as an anchor for the growing polyketide chain [39]. In aurachin biosynthesis, the AuaB-bound anthranilic acid is condensed with two malonyl-CoA units. AuaC acts as keto synthase (KSα), catalyzing the iterative Claisen condensation of the malonyl-CoA units with the substrate. AuaD is the KSβ or chain length factor (CLF) involved in the decarboxylation of the substrate and determination of the chain length. After the condensation of anthranilic acid with two malonyl-CoA units, the resulting 4-hydroxy-2-methylquinoline is released by the PKS and tautomerizes to 2-methyl-1*H*-quinolin-4-one [37,38]. This intermediate is then prenylated with a farnesyl moiety by the membrane-bound prenyltransferase AuaA to produce **1** [41]. The next step in aurachin biosynthesis is the *N*-hydroxylation of **1** to **2** by the Rieske monooxygenase [2Fe-2S] AuaF [38]. The relocation of the farnesyl side chain from position 3 to position 4 yields aurachin B (**3**). For this, the flavin-dependent monooxygenase AuaG prepares the relocation of the side chain by a C2-C3 epoxidation followed by an acid-base catalyzed ring opening. Afterward, AuaG repositions the side chain by a semipinacol rearrangement or a retro-[2,3]-Wittig and Claisen rearrangement and AuaH catalyzes the reduction to **3** [38,42,43]. Finally, the flavin-dependent monooxygenase AuaJ catalyzes a C2′-C3′ epoxidation of the farnesyl moiety in **3**. The putative steroid δ-isomerase AuaI supports the opening of the epoxide by a proposed push-pull mechanism leading to a nucleophilic attack at position 2′ from the OH-3. This results in the C2′-O-4 cyclization and the 3′-hydroxylation of the farnesyl moiety forming **4** [38].

**2** and **4** are the primary products of the aurachin biosynthetic pathway, whereas **1** and **3** are minor metabolites [34]. The biosynthesis of a large number of other aurachin derivatives of *S. aurantiaca* Sg a15 is still not clear, and it was speculated that the corresponding compounds might originate from non-specific reactions of house-keeping enzymes or spontaneous (i.e., non-enzymatic) reactions [38].

**Figure 3:**
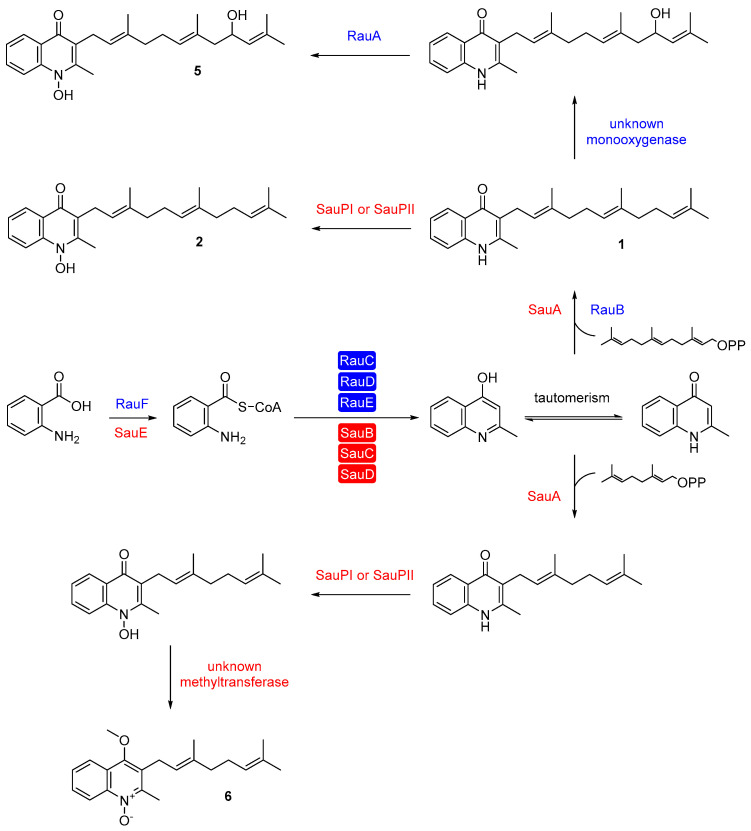
Biosynthesis of aurachin RE (**5**) in *R. erythropolis* JCM 6824 (blue enzymes) and biosynthesis of aurachin SS (**6**) in *Streptomyces* sp. NA04227 (red enzymes). Boxed enzymes are part of a type II PKS.

Aurachin biosynthesis was initially explored in *S. aurantiaca* Sg a15. Its subsequent analysis in other producer organisms revealed some unexpected differences. In the actinomycete *R. erythropolis* JCM 6824, for instance, one enzyme is responsible for activating and loading anthranilic acids to the acyl carrier protein RauC, namely the anthranilate-CoA:ACP transferase and anthranilate-CoA ligase RauF (Figure 3) [44]. In the next step, the PKS system (RauCDE) condenses two malonyl-CoA units with the substrate, analogous to the biosynthesis in *S. aurantiaca* Sg a15. The PKS of *Rhodococcus* also produces 4-hydroxy-2-methylquinoline as an intermediate, which is prenylated by the membrane-bound farnesyltransferase RauB to form **1** [44]. The last two steps towards aurachin RE (**5**) involve an unknown monooxygenase and the cytochrome P450 monooxygenase RauA [44,45]. Furthermore, the organization of the aurachin biosynthesis genes differs in *S. aurantiaca* and *R. erythropolis*. In the latter bacterium, all genes required for aurachin assembly are clustered in one single locus. In contrast, the corresponding open reading frames are distributed over three remote loci in the genome of *S. aurantiaca* [38,44]. Moreover, only three genes in the two producer organisms show sequence homology to each other (*rauB*—*auaA*, *rauD*—*auaC*, and *rauF*—*auaE*). *Rhodococcus* has additional genes for producing the farnesyl moiety (*rauH* and *rauI*) as part of its aurachin gene cluster and uses a cytochrome P450 monooxygenase instead of a Rieske monooxygenase [2Fe-2S] for the *N*-hydroxylation of the quinolone nucleus [44].

**Figure 4:**
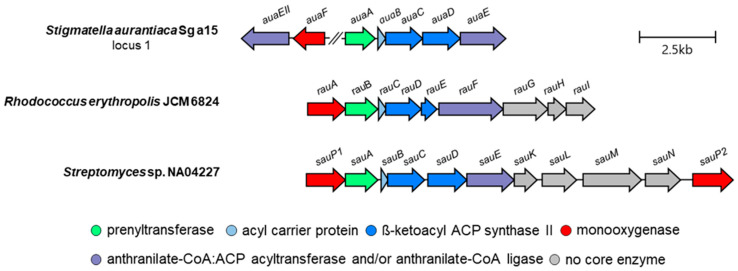
Organization of the aurachin gene clusters in *S. aurantiaca* Sg a15 (loci 2 and 3 containing *auaG*—*auaI* are not displayed), *R. erythropolis* JCM 6824, and *Streptomyces* sp. NA04227. Genes of isofunctional enzymes of the core aurachin biosynthesis are highlighted in the same color.

The biosynthesis of aurachins in *Streptomyces* sp. NA04227 is highly similar to that in *R. erythropolis* JCM 6824 (Figure 3). A single enzyme (SauE) catalyzes the activation of anthranilic acid and the loading to the acyl carrier protein (SauB). At the same time, the *N*-hydroxylation of the quinolone nucleus is carried out by a cytochrome P450 monooxygenase (SauPI or SauPII) [12]. Unlike AuaA and RauB, the farnesyltransferase SauA also utilizes geranyl pyrophosphate for the prenylation reaction. This results in geranylated derivatives of **1** and **2**. Subsequently, the derivative of **2** is converted by an unidentified methyltransferase to aurachin SS (**6**). Another unique feature of the aurachin biosynthesis gene cluster in *Streptomyces* sp. NA04227 is the presence of a thioesterase gene. The putative thioesterase SauK might contribute to the chain release from the type II PKS and the following cyclization [12]. An overview of the aurachin cluster architectures and their known and predicted gene functions are presented in Figure 4 and Table 1. 

## 4. Biotechnological Production and Derivatization of Aurachins

Aurachin D (**1**) might become a promising drug candidate for the treatment of bacterial and protozoal infections and it already represents a valuable pharmacological tool compound [15,27]. However, it is only produced as a minor metabolite in *S. aurantiaca* [34]. To secure the production of **1**, the reconstruction of its biosynthesis in a heterologous host was attempted to increase the titer and facilitate the downstream processing. Initially, *R. erythropolis* JCM 3201 was evaluated as a host organism for recombinant aurachin production. However, no titers were reported, and the production was accompanied by growth defects, which were attributed to the potent antibiotic activity of aurachins [44]. Besides toxicity, the assembly of the required PKS multienzyme complex, the activation of the ACP, and the prenylation pose further challenges for recombinant aurachin production. The ACP needs to be activated by the posttranslational attachment of a phosphopantetheinyl arm to a conserved serine residue. A phosphopantetheinyl transferase (PPtase) is required for this reaction [46]. PPtases differ in their substrate specificity, which is why a potential host for aurachin production must provide a PPtase that recognizes the ACP of the aurachin PKS [47]. The prenylation reaction in aurachin biosynthesis is carried out by a membrane-bound enzyme [12,41,44]. The heterologous expression of membrane-bound enzymes is usually difficult. It can be hindered by the accessibility of the ribosomal binding site, the oversaturation of the translocation system, or the disruption of the natural membrane leading to toxicity effects [48].

The aforementioned challenges were recently overcome with the biotransformation of 2-methyl-1*H*-quinolin-4-one to aurachin D in *E. coli* [49]. Although aurachin D is an extremely potent inhibitor of *E. coli* cytochrome *bd* oxidases, toxicity-associated effects can be reduced or even completely avoided if the heterologous host is grown under aerobic, oxygen-rich conditions [21,22]. The requirement of expressing the aurachin PKS and a PPtase with broad substrate specificity was circumvented by reconstructing the biosynthesis from 4-quinolones and not from anthranilic acid. This approach was feasible because the chemical synthesis of 4-quinolones is well-established and concise [50]. To achieve a high-level production of the membrane-bound prenyltransferase AuaA, the expression of the corresponding *auaA* biosynthesis gene was translationally coupled to an upstream cistron following a bicistronic design (BCD) strategy [49]. The BCD mimics the operon architecture found in many bacterial gene clusters. For this purpose, the start codon of the gene of interest is nearby or overlapping with an upstream cistron of a short leader peptide [51]. The translation of the upstream cistron can then resolve downstream secondary structures in the mRNA blocking the accessibility of the downstream cistron due to the helicase activity of the ribosome [52]. A library of BCD elements with varying translation initiation strength was screened to find the optimal expression strength for *auaA* [49]. The aurachin titer of the recombinant host could be further raised by codon-optimization of *auaA* and by improving the cellular supply of farnesyl pyrophosphate through the introduction of the mevalonate pathway. Ultimately, the titer could be raised to 17.0 mg L^−1^ [49], which exceeded the reported titer (<1 mg L^−1^) of the natural producer *S. aurantiaca* [8]. 

Derivatization is a powerful tool to improve the activity of a natural product and obtain new insights into its target site. Generally, chemical synthesis is employed for the derivatization of natural products [53] and, as a matter of fact, some of the reported synthesis routes for aurachins were also used to generate new aurachin analogs [14,18,27]. However, all established chemical syntheses require the installation of the farnesyl side chain before the quinolone heterocycle is formed. This prevents the use of 4-quinolone analogs as starter compounds, thereby increasing the complexity of the chemical derivatization process of aurachins. For the biotechnological derivatization of aurachins, three different approaches were pursued. Precursor-directed biosynthesis was used to introduce halogenated anthranilic acids into the aurachin pathway [54]. However, this approach had some drawbacks. Even though only a single substrate surrogate was provided in every feeding experiment, a number of aurachin derivatives were produced, which complicated the purification of these compounds. Moreover, only fluorinated analogs of **1** were generated in sufficient quantities [54]. Another approach involved the in vitro biotransformation of different 4-quinolone analogs using the prenyltransferase AuaA from *S. aurantiaca* Sg a15 [41]. In contrast to the previously described in vivo biotransformation, this approach required the addition of farnesyl pyrophosphate to the reaction mix. Since farnesyl is a relatively expensive and unstable substrate, the most recent approach relied on the in vivo biotransformation of different 4-quinolone analogs. For this, the previously described *E. coli* production strain was used [15].

## 5. Conclusions

Aurachins are a remarkable class of natural products. They are already valuable tool compounds, which have helped to gain important structural and functional insights into bacterial respiratory chains [21,29,30], which can be clearly discriminated from their eukaryotic counterparts [55]. The aurachins are also exciting starting points for developing new respiratory chain inhibitors or potential drugs against infectious diseases. In combination with drugs acting on different sites of the respiratory chain, these antibiotics might become highly effective therapeutic agents [27,56]. The development of aurachin-derived antibacterial drugs is, however, still hindered by unspecific cytotoxic effects and poor drug delivery [14,27]. Nano-based drug delivery might solve both problems. For example, lipid nanoparticles can fuse with the bacterial cell wall and directly provide the antibiotic to the target bacteria [57]. Thereby, antibiotics such as **1** can potentially overcome the outer membrane of a Gram-negative bacterium and are only active at the target site. This may also reduce cytotoxic effects for the patient. Furthermore, nanocarriers can protect an antibiotic from enzymatic degradation and prolong its half-time [57].

Especially **1** showed potent bioactivity against *Leishmania donovani*, the pathogen of visceral leishmaniasis [15]. Although the exact mechanism behind the antileishmanial activity and the in vivo efficacy of **1** need to be further investigated, an impairment of the parasite’s electron transport processes seems plausible. The leishmanial respiratory chain and, in particular, NDH2 have become promising molecular targets [58]. As opposed to *P. falciparum* [59], NDH2 was found to be vital for *Leishmania*, including parasites with an active complex I NADH dehydrogenase [60]. Considering that an analog of **2** was reported as a potent inhibitor of NDH2 in the rodent malaria parasite *Plasmodium yoelii yoelii* [33], one might assume that **1** also exerts its antileishmanial effects, at least partially, via NDH2 inhibition. Recently, the ubiquinone reduction site of cytochrome *b*, part of the cytochrome *bc1*, was demonstrated to be a promiscuous drug target in *T. cruzi* and *L. donovani* [61]. Early studies on the cytochrome *bc*_1_ of *Rhodospirillum rubrum* suggested that the ubiquinone reduction site is targeted by **2** and the oxidation site by **1**. However, the inhibition of the reduction site by **1** could not be excluded [17]. Of course, **1** could also affect additional target sites in *L. donovani*.

Besides elucidating the antileishmanial mode of action of aurachins, another prerequisite for their preclinical development is to secure a sufficient supply of these antibiotics. Some progress has been achieved by successfully establishing the heterologous production of **1** [49]. However, the titer needs further improvement. Protein engineering, optimization of the culture condition, or strain optimization are possible solutions that should be explored. Particularly, the metabolic engineering of the farnesyl supply is a valid target to increase the titer of **1** in *E. coli*. Because gram-scale heterologous production of different isoprenoids is possible in *E. coli* [62], carbon flow in the aurachin production might not be optimal adjusted to supply the required farnesyl moiety for the prenylation of the fed substrate.

## Figures and Tables

**Table 1:** Genes of the aurachin biosynthesis gene clusters in *S. aurantiaca* Sg a15 (A), *R. erythropolis* JCM 6824 (B), and in *Streptomyces* sp. NA04227 (C) as well as the known or predicted functions of their products.

A	Function of Gene Product	B	Function of Gene Product	C	Function of Gene Product
*auaA*	prenyltransferase	*rauA*	cytochrome P450 monooxygenase	*sauA*	prenyltransferase
*auaB*	acyl carrier protein	*rauB*	prenyltransferase	*sauB*	acyl carrier protein
*auaC*	β-ketoacyl ACP synthase II (KSα)	*rauC*	acyl carrier protein	*sauC*	β-ketoacyl ACP synthase II
*auaD*	β-ketoacyl ACP synthase II (KSβ)	*rauD*	β-ketoacyl ACP synthase II	*sauD*	β-ketoacyl ACP synthase II
*auaE*	anthranilate-CoA:ACP acyltransferase	*rauE*	β-ketoacyl ACP synthase II	*sauE*	anthranilate-CoA:ACP acyltransferase and anthranilate-CoA ligase
*auaEII*	anthranilate-CoA ligase	*rauF*	anthranilate-CoA:ACP acyltransferase and anthranilate-CoA ligase	*sauK*	thioesterase
*auaF*	Rieske [2Fe–2S] monooxygenase	*rauG*	efflux protein	*sauL*	*trans*-isoprenyl diphosphate synthase
*auaG*	flavin-dependent monooxygenase	*rauH*	isopentenyl diphosphate isomerase	*sauM*	DXP synthase (MEP pathway)
*auaH*	reductase	*rauI*	farnesyl synthase	*sauN*	HMBDP synthase (MEP pathway)
*auaJ*	flavin-dependent monooxygenase			*sauPI*	cytochrome P450 monooxygenase
*auaI*	steroid δ-isomerase			*sauPII*	cytochrome P450 monooxygenase

## Data Availability

Not applicable.
